# Effectiveness of interventions to improve medication adherence in adults with depressive disorders: a meta-analysis

**DOI:** 10.1186/s12888-022-04120-w

**Published:** 2022-07-20

**Authors:** Beatriz González de León, Tasmania del Pino-Sedeño, Pedro Serrano-Pérez, Cristobalina Rodríguez Álvarez, Daniel Bejarano-Quisoboni, María M. Trujillo-Martín

**Affiliations:** 1 Unidad Docente Multiprofesional de Atención Familiar y Comunitaria “La Laguna ‑ Tenerife Norte”, Gerencia de Atención Primaria del Área de Salud de Tenerife, Santa Cruz de Tenerife, Spain; 2 Fundación Canaria Instituto de Investigación Sanitaria de Canarias (FIISC), Santa Cruz de Tenerife, Spain; 3 Servicio de Evaluación y Planificación del Servicio Canario de La Salud, Santa Cruz de Tenerife, Spain; 4 Servicio de Psiquiatría, Hospital Universitario Vall d’Hebron, Barcelona, Spain; 5 Departamento de Psiquiatría Y Medicina Legal, Universidad Autónoma de Barcelona, Barcelona, Spain; 6 Grupo de Investigación en Psiquiatría, Salud Mental Y Adicciones, Vall d’Hebron Instituto de Investigación (VHIR), Barcelona, Spain; 7 Campus Ciencias de La Salud. Área de Medicina Preventiva y Salud Pública. Universidad de La Laguna, Santa Cruz de Tenerife, Spain; 8 Centro Superior de Investigación en Salud Pública (CSISP-FISABIO), Valencia, Spain; 9 Red de Investigación en Servicios de Salud en Enfermedades Crónicas (REDISSEC), Madrid, Spain

**Keywords:** Major Depressive Disorder, Meta-analysis, Systematic review, Treatment Adherence

## Abstract

**Background:**

Non-adherence to medication is a major obstacle in the treatment of depressive disorders. We systematically reviewed the literature to evaluate the effectiveness of interventions aimed at improving adherence to medication among adults with depressive disorders with emphasis on initiation and implementation phase.

**Methods:**

We searched Medline, EMBASE, The Cochrane Central Register of Controlled Trials (CENTRAL), PsycINFO, Social Science Citation Index and Science Citation Index for randomized or non-randomized controlled trials up to January 2022. Risk of bias was assessed using the criteria of the Cochrane Collaboration. Meta-analyses, cumulative and meta-regression analyses for adherence were conducted.

**Results:**

Forty-six trials (*n* = 24,324) were included. Pooled estimate indicates an increase in the probability of adherence to antidepressants at 6 months with the different types of interventions (OR 1.33; 95% CI: 1.09 to 1.62). The improvement in adherence is obtained from 3 months (OR 1.62, 95% CI: 1.25 to 2.10) but it is attenuated at 12 months (OR 1.25, 95% CI: 1.02 to 1.53). Selected articles show methodological differences, mainly the diversity of both the severity of the depressive disorder and intervention procedures. In the samples of these studies, patients with depression and anxiety seem to benefit most from intervention (OR 2.77, 95% CI: 1.74 to 4.42) and collaborative care is the most effective intervention to improve adherence (OR 1.88, 95% CI: 1.40 to 2.54).

**Conclusions:**

Our findings indicate that interventions aimed at improving adherence to medication among adults with depressive disorders are effective up to six months. However, the evidence on the effectiveness of long-term adherence is insufficient and supports the need for further research efforts.

**Trial registration:**

International Prospective Register for Systematic Reviews (PROSPERO) number: CRD42017065723.

**Supplementary Information:**

The online version contains supplementary material available at 10.1186/s12888-022-04120-w.

## Introduction

Depression is a common mental disorder typically chronic, disabling and frequently comorbid that affects more than 260 million people every year [1] and causes considerable personal suffering and has great economic costs for Western societies [2]. Depression was expected to be the leading cause of disability in 2030 [3] but, as early as 2021, it was declared the leading cause of disability worldwide and a major contributor to the overall global burden of disease according to the World Health Organization [4].

Although pharmacological treatment of depressive disorders has shown a considerable efficacy, patients do not always take their medication as instructed. When talking about the behaviors of patients in taking medication, adherence and persistence need to be examined.

Medication adherence can be defined as the process to which a patient acts within the prescribed range and dose of a dosage regimen, described by three quantifiable phases: 1) initiation, when patient takes the first dose; 2) implementation, defined as the process to which a patient's actual dosing corresponds to the prescribed dosing regimen; and 3) discontinuation, when the next dose to be taken is omitted and no more doses are taken thereafter [5]. Persistence refers to the duration of time from initiation to discontinuation of therapy [5]. In this sense, non-adherence to appropriately prescribed medicines remains a major challenge in current clinical psychiatric practice that compromises the efficacy of available treatments and interferes with patient recovery [6].

The impact of non-adherence to antidepressants increases the likelihood of relapse and/or recurrence, emergency department visits, and hospitalization rates; increases symptom severity and decreases treatment response and remission rates [7]. Non-adherence subsequently translates to an increase in medical and total healthcare utilization [7]. Available literature shows primary medication adherence (when a patient properly fills the first prescription for a new medication) rates ranging between 74 and 82% [8, 9], but unfortunately, approximately 50% of patients prematurely discontinue therapy [10, 11].

Socio-demographic variables, such as age, positive attitudes to prescribed medication and previous experiences were found to be factors predicting better adherence in patients with depressive disorders. Conversely, experience of side effects, dissatisfaction with treatment and a poor patient–professional relationship were found to be associated with poorer adherence [12].

Several interventions have been designed to improve medication adherence. Some evidence suggests that multifaceted interventions targeting the patient, physician and structural aspects of care are more effective than single-component interventions [13–15]. However, it is considered that intervention strategies should be designed to address the specific factors associated with non-adherence to psychotropic medication for each psychiatric disorder [16, 17]. Moreover, interventions rarely target the adherence phase but recruit patients independently of their treatment journey that is, at the beginning (initiation), during implementation or while discontinuing (persistence) [18].

The aims of the present study are to identify, critically assess and synthesize the available scientific evidence on the effectiveness of interventions aimed at improving adherence (initiation and the implementation phase) to medication among adults with depressive disorders.

## Material and methods

A systematic review and meta-analysis were performed according to the Cochrane Handbook [19] and reported in accordance to Preferred Reporting Items for Systematic Reviews and Meta-Analyses (PRISMA) guidelines [20]. The protocol of the present review was registered in Prospero (CRD42017065723).

### Information sources and search strategy

The following electronic databases were searched (January 2022): Medline (OVID interface), EMBASE (Elsevier interface), CENTRAL (The Cochrane Library interface), PsycINFO (EBSCO interface), SCI-EXPANDED (Web of Science interface) and SSCI (Web of Science interface). The search strategy was initially developed in Medline, using a combination of controlled vocabulary and free text terms and was then adapted for each of the other databases. Search terms included the following: depressive disorder, medication and adherence. Searches were limited to the English and Spanish languages and no date restriction was imposed. The full search strategy is available in Supplementary Material (see Supplementary Table 1). The reference lists of all included papers were also examined to identify possible additional studies meeting selection criteria.

### Selection criteria

Studies were eligible for inclusion if they fulfilled the following criteria: 1) randomized controlled trials (RCTs) or non-randomized controlled trials (nRCTs), with allocation of both individuals and clusters; 2) any type of intervention (whether they were psychotherapeutic, educational interventions or other clinical intervention such as monitoring and adjustment of pharmacological treatment) aimed at increasing adherence (initiation and/or implementation phase) to anti-depressive medication administered to adults (18–65 years) with a diagnosis of depressive disorder. If a study addressed a heterogeneous group of patients, the study was included as long as the results for patients meeting the inclusion criteria were reported separately or they accounted for more than 80% of the target population. If the phase of adherence was not specified according to the taxonomy of Vrijens et al. [5], the reviewers determined the phase in which the evaluation was carried out based on the characteristics described in the study (adherence measurement method and moment); 3) usual care or alternative intervention as comparison group; 4) studies assessing initiation or implementation phase divided into three temporary spaces: short-term (closest to 3 months), medium-term (closest to 6 months) or long-term (closest to 12 months) adherence to prescribed medication; 5) studies published in English or Spanish. Exclusion criteria included: 1) studies examining patients with bipolar depression or schizoaffective disorder, and 2) studies with fewer than 10 study participants.

### Study selection process

Two reviewers addressed eligibility independently and in duplicate. Firstly, the title and abstract of references identified in the electronic search were screened. Secondly, the full text of the studies that appeared to fulfil the pre-specified selection criteria was read and evaluated for inclusion. Disagreements between reviewers were resolved through discussion with the research team until consensus was reached.

### Data collection process

A data extraction form was prepared by the authors, pilot tested on two studies and refined accordingly. One reviewer extracted the following data from the included studies: identification of the article (author, date of publication, country), study objective and methodology (design, context, duration), details of participants (selection criteria and demographics), interventions (type, modality and number of sessions), comparators and outcome (adherence definition, measurement method and value), and finally results. A second reviewer subsequently verified the extracted data. When any required information was missing or unclear in a paper, an effort was made to contact the corresponding author.

### Risk of bias assessment

Two reviewers independently and in duplicate assessed risk of bias of included studies using the Cochrane Risk of Bias tools for RCT (RoB 2.0) [21] with the additional guidance for cluster-RCT [22] and nRCT (ROBINS-I) [23]. Discrepancies of judgments between the reviews were discussed by the research team until consensus was reached.

### Assessment of publication bias

According to the recommendations of the Cochrane Collaboration [19], the presence of publication bias was assessed considering the size and sponsorship of the included studies, and by constructing a funnel plot and computing the Egger’s regression test using metafunnel and metabias commands in STATA version 14, respectively.

### Analysis and synthesis of results

Meta-analyses and forest plots were performed for the adherence rate using the metan commands in STATA version 14. Effects of interventions were estimated as odd ratios (OR), with 95% confidence intervals (CI). Heterogeneity was assessed using the I^2^ statistic. When there was heterogeneity (I^2^ ≥ 25%), meta-analyses were performed using a random-effects model using the method of DerSimonian and Laird and taking the estimate of heterogeneity from the Mantel–Haenszel model. When there was neither clinical nor statistical heterogeneity, a fixed-effect model was used [24].

Several sources of heterogeneity relating to the characteristics of the study population and the interventions were anticipated. Predictive variables included age, gender, diagnoses, type of intervention, providers of the intervention (multidisciplinary vs. non-multidisciplinary team), modality of intervention (face-to-face vs. telephone, mails and/or website) and number of sessions. When reported in most studies, the effect of these study-level variables on the effectiveness closest to six months after intervention using subgroup analyses (diagnoses, type of intervention, providers of intervention and modality of intervention) and meta-regression techniques (age, gender, and number of sessions) were explored using the metareg command.

Sensitivity analyses were conducted to assess the stability of the effects of excluding certain types of studies (n-RCT).

Cumulative meta-analysis was used to evaluate the sufficiency and stability over time of the effects of interventions aimed at increasing adherence to anti-depressive medication. Studies were sequentially added by year of publication to a random- effects model using the metacum user-written command.

## Results

**Fig. 1:**
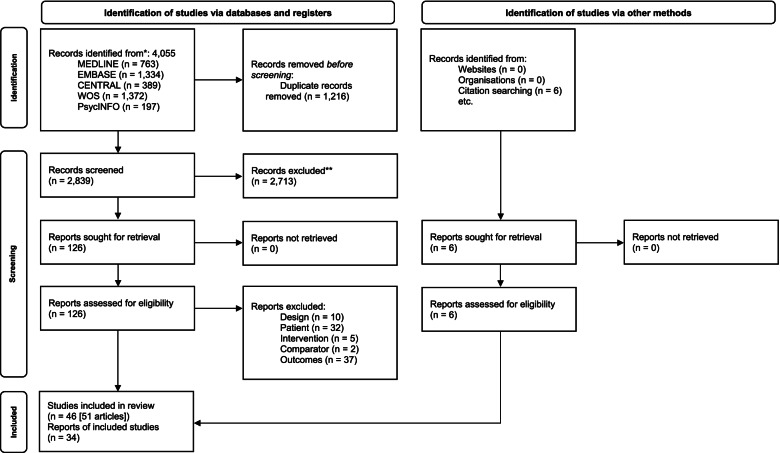
Flow diagram of the selection process of studies

Out of a total of 2,839 initially identified references after eliminating duplicates, 40 studies were selected after full-text screening (Fig. 1). The manual search provided six additional studies, thus, 46 studies (published in 51 papers) were finally eligible for inclusion according to the pre-established selection criteria [25–75].

### Characteristics of included studies

The 46 included trials were published in English between 1976 and 2021 (Table 1). Thirty-four are individual-RCT [25, 29–36, 40, 42–44, 46, 48–52, 55–61, 64–67, 70, 71, 74, 75], seven are cluster-RCT [26, 38, 41, 52, 53, 63, 72], four are individual-nRCT [28, 39, 45, 47], and one is cluster-nRCT [27]. The duration of reported follow-up ranged from 4 to 76 weeks (median 32 weeks). Seven studies specified incentive payments to patients [27, 29, 38, 39, 46, 55, 61] and 43 of them were carried out in outpatient [25, 26, 28–43, 46–74].

**Table 1:** Main characteristics of included studies

Study Country	Design	Follow-up (w)	Sample							Intervention					Outcome	
			**Size**			**Age (years) Mean, (SD)**	**Gender (female) (%)**	**Diagnoses**	**Inclusion Criteria**	**Type**	**Modality**	**Nº of sessions**	**Duration (m)**	**Staff qualification**	**Measure**	**Period (w)**
			**N**	**IG**	**CG**											
Adler et al., 2004 USA [25]	RCT	16	533	268	265	42.3 (13.9)	71.80	MDD ± PDD	≥ 18 years MDD and/or PDD (DSM-IV) English reading comprehension	CCM	Face-to-face	9	6	Doctoral-level clinical pharmacist	Correct medication intakes	Base 12 24
Akerblad et al., 2003 Sweden [26]	Cluster RCT	24	1,031	366	339	48.4 (14.36)	28.10	MDD	≥ 18 years MDD (DSM-IV) SSRI prescription	Education + support (programme RHYTHMS)	Letters + telephone	5 letters + 4 telephone calls	6	GPs	Self-report	24
															Serum levels	24
															Appointments kept	24
															Composite index	24
Aljumah and Hassali, 2015 Saudi Arabia [59]	RCT	16	239	119	120	39.5 (NR)	58.16	MDD	18–60 years MDD (DSM-IV) AD prescription	SDM	Face-to-face	2	6	Pharmacist, psychiatrist and trained nurse	MMAS	12
Al-Saffar et al., 2008, 2005 Kuwait [37]	RCT	20	300	100	100	NR	33.10	MDD	≥ 18 years Unipolar depression (ICD-10) TCA or SSRI prescription	Counselling	Face-to-face + leaflet	1	NA	Trained pharmacist	Self-report + Pill count	20 6
				100						Education + support	Leaflet				Correct medication intakes	6
Browne et al., 2002 Canada [70]	RCT	24	707	212	196	42.4 (NR)	68.00	PDD ± MDD	18–75 years PDD ± MDD (DSM-IV)	Interpersonal psychotherapy	Face-to-face	10	6	Masters-level therapist	Correct medication intakes	24
Capoccia et al., 2004 USA [71]	RCT	52	74	41	33	38.7 (13.5)	57.00	Depressive episode	≥ 18 years Depressive episode New AD prescription	CCM	Telephone	16	12	Clinical pharmacist	Self-report	12 24 36 52
Chang et al., 2014 USA [72]	Cluster RCT	24	915	503	411	46.03 (21.49)	66.30	MDD	≥ 18 years MDD Newly prescribed AD Capable of self-management and understand English	Monitoring and feedback to physicians about the patient's symptom severity	Telephone	6	6	GPs or internal medicine doctors	Correct medication intakes and adapted questions from MMAS	12 24
de Jonghe et al., 2001 Netherlands [74]	RCT	24	167	83	84	34 (19–60)	62.00	PDD ± MDD	18–60 years DSM-III criteria MDD with or without dysthymia 17-item HDRS ≥ 14 Written informed consent	Short Psychodynamic Supportive Psychotherapy	Face-to-face	16	6	Psychiatrist ± fully trained psychotherapist	Pharmacotherapy dropout rates	24
Desplenter et al., 2013 Belgium [27]	Cluster nRCT	52	99	41	58	46.10 (11.10)	62.60	MDD	≥ 18 years; MDD ( DSM-IV-TR) AD prescription Dutch speaking Could be reached by telephone for follow-up	Tailoring counselling or counselling intervention	Telephone	1	1 day	Pharmacist	MMAS	4 12
Gervasoni et al., 2010 Switzerland [28]	nRCT	2	131	81	50	36.24 (19–62)	59.54	Moderate or severe depressive episode	18–65 years; Moderate or severe depressive episode without psychotic characteristics (ICD-10) MADRS scale ≥ 25	Monitoring and motivational support	Telephone	3	2 weeks	Psychiatrist and research nurse	AD plasma level	2
Guo et al., 2015 China [65]	RCT	24	81	44	37	41.10 (12.10)	64.16	Moderate to severe MDD	Outpatients 18–65 years Non-psychotic MDD ( DSM-IV) HAM-D ≥ 17	Measurement-based care	Face-to-face	NA	NA	Psychiatrist and raters	NR	12
Hammonds et al., 2015 USA [29]	RCT	4	57	30	27	20.6 (4.3)	85.96	MDD (89,4%)	18–30 years AD prescription English speaking Patients who had an Android or iPhone smartphone	Medication reminder app	Smartphone	Until study termination	1	Trained research assistant	Correct medication intakes	4
Interian et al., 2013 USA [30]	RCT	20	50	26	24	40.6 (16.90)^a^	76.00	MDD or PDD	≥ 18 years MDD or PDD (DSM-IV) AD prescription	Motivational Enhancement Therapy	Face-to-face	3	5	Clinical psychologist and psychology doctoral students	Pill Count	5 20
John et al., 2016 India [31]	RCT	6	39	17	22	34 (21–46)	61.53	Mild depression, moderate depression or PDD	18–60 years Depression or PDD (ICD-10) AD monotherapy 12–23 HAM-D score	Educational	Face-to-face	NR	NR	Clinicians	Correct medication intakes	6
Katon et al., 2002 USA [32]	RCT	112	171	NR	NR	46.95 (18–80)	74.55	MDD	18–80 years; new AD prescription ≥ 11 SCL-20 and > 4 DSM-IV or < 4 DSMIV and ≥ 11,5 SCL-20	CCM	Face-to-face	0–7	28	GPs and psychiatrist	Adequate prescription refills	24 48 72 96 112
Katon et al., 2001 USA [33]	RCT	52	386	194	192	46.0 (17.85)^a^	73.70	MDD or PDD	18–80 years MDD or PDD AD prescription	CCM	Mail + website	4 mailings + 3 telephone calls	12	GPs, psychologists, nurse practitioners and social worker	Automated data on refill	12 24 36 52
Katon et al., 1999 USA [34]	RCT	24	228	114	114	46.9 (19.38)^a^	74.50	MDD or PDD or anxiety	18–80 years MDD or PDD ≥ 4 DSM- III-R major depressive symptoms + SCL-20 score ≥ 1.0 or < 4 major depressive symptoms + SCL-20 score ≥ 1.5	CCM	Book + videotape + face-to-face	≤ 7	6	GPs and psychiatrist	Automated data on refill	4 12 24
Katon et al., 1996 USA [35]	RCT	12	153	31	34	44.4 (26.88)^a^	73.86	MDD	18–75 years Definite or probable MDD or PDD SCL-20 score ≥ 0.75 Willingness to take AD	CCM	Book + videotape + face-to-face	4–6 sessions + 4 telephone calls	6	GPs and psychologist	Automated data on refill	4 12
Katon et al., 1995 USA [36]	RCT	12	217	108	109	35.9 (28.83)^a^	77.60	MDD or PDD	18–780 years Definite or probable MDD or PDD SCL-20 score ≥ 0.75 Willingness to take AD	CCM	Book + videotape + face-to-face	4	1	GPs, therapists and psychiatrists	Automated data on refill	4 12
Keeley et al., 2014 USA [38]	Cluster RCT	NR	175	85	86	33.40 (38–60)	38.05	Depression	≥ 18 years newly diagnosed English speakers Consenting patients Positive Patient Health Questionnaire ≥ 10 PHQ-9 score	Motivational Interviewing	Face-to-face	4	13	GPs	NR	
Klang et al., 2015 Israel [39]	nRCT	24	NR	173	12,746	50.5 (25.96)^a^	68.05	Depressive episode	≥ 18 years Depressive episode (DSM-IV) Escitalopram prescription	Pharmacist adherence support	Face-to-face	6	NR	Community Pharmacist	Correct medication intakes	4 24
Kutcher et al., 2002 Canada [40]	RCT	29	269	131	138	NR	NR	MDD	MDD (DSM-IV) Contraceptive method in females of childbearing years	Education + support (programme RHYTHMS)	Letters + telephone	5 letters + 4 telephone calls	6	Research nurses	Pill count	NR
LeBlanc et al., 2015 USA [41]	Cluster RCT	24	297	138	139	43.5 (43.54)^a^	66.92	Moderate to severe depression	≥ 18 years Moderate/Severe depression PHQ-9 score ≥ 10	SDM	Face-to-face	2	6	Clinicians	Automated data on refill	24
Lin et al., 2003 USA [42]	RCT	52	386	194	192	46.0 (17.85)^a^	26.40	High risk for recurrent depression	18–80 years AD prescription Improvement of depressive episode (≥ 4 DSM- III-R major depressive symptoms or 4 major depressive symptoms + SCL-20 score ≥ 1.5) High risk of relapse (≥ 3 lifetime depressive episodes or a history of dysthymia)	CBT + motivational interviewing + education	Face-to-face + telephone	2 sessions + 3 telephone calls	12	Psychologist, psychiatric nurse and social worker	% of days covered	52
Lin et al., 1999 USA [43]	RCT	19	156	63	53	44.10 (13.60)	81.00	MDD	18–80 years AD prescription SCL-20 score ≥ 0.75	CCM	Face-to-face	4 + 2 optional	4.75	GPs and psychologists	Self-reported and adequate pharmacotherapy according to pharmacy data	19
Mantani et al., 2017 Japan [44]	RCT	17	164	81	83	40.90 (NR)	53.05	MDD ± anxiety	25–59 years; MDD without psychotic features (DSM-5 and PRIME-MD); antidepressant-resistant, BDI-II ≥ 10 for ≥ 4 weeks; AD in monotherapy (not antipsychotics or mood stabilizers); smartphones users; being an outpatient; no plan to transfer within 4 months	Smartphone CBT	Smartphone	8	2.25	Psychiatrists	Discontinuation of protocol antidepressant treatment by week 9	17
Marasine et al., 2020 Nepal [69]	RCT	16	196	98	98	NR	142 (72,45)	Depression	18–65 year Diagnosed with depression AD prescription	Education + support	Face-to-face + leaflet	1	NA	Clinical pharmacist	MMAS	16
Meglic et al., 2010 Slovenia [45]	nRCT	24	19	10	9	35.71 (12.11)	86.00	Depression or mixed anxiety and depression disorder	ICD10 group F32 or F41.2 first time or after a remission > 6 months Newly AD Internet and mobile phone BDI-II ≥ 14	CCM	Telephone + website	NR	6	GPs and psychologist	Correct medication intakes	24
Mundt et al., 2001 USA [46]	RCT	30	246	124	122	40.5 (16.57)^a^	82.83	MDD	MDD (DSM-IV) Symptom duration of ≥ 1 month AD prescription Hamilton Depression score ≥ 18	Education + support (programme RHYTHMS)	Mail + telephone	1mailing + telephone calls	7	NR	Medication days	30
Myers and Calvert, 1984 UK [49]	RCT	NR	120	40	40	41.7 (29.79)	74.20	Depression	Depression, reactive or endogenous Dothiepin prescription	Education	Leaflet	1	NA	NA	Correct medication intakes	3 6
Myers and Calvert, 1976 UK [47]	nRCT	NR	89	46	43	47.8 NR	66.30	Depression	21–77 years ≥ Attack of primary depression, reactive or endogenous Dothiepin prescription	Education	Leaflet	1	NA	NA	Correct medication intakes	NR
Nwokeji et al., 2012 USA [50]	RCT	52	166	101	65	47.8 (12.01)^a^	88.00	MDD	MDD AD prescription	Enhanced care	Mail + telephone	NR	12	Nurses and social worker	% of days covered	52
Perahia et al., 2008 11 European countries [51]	RCT	4	962	485	477	46.2 (18.46)^a^	64.20	MDD	≥ 18 years MDD (DSM-IV) Hamilton Depression score ≥ 15 Access to a telephone	Education	Telephone	3	12	GPs or psychiatrists	Pill count	2 6 12
Perlis et al., 2002 USA [75]	RCT	28	132	66	66	39.9 (14.57)^a^	54.60	MDD	18–65 years MDD (DSM-III-R) Hamilton Depression score ≥ 16 History of ≥ 3 major depressive episodes, diagnosis of current episode as chronic; history of poor interepisode recovery; or both MDD and PDD	CBT	Face-to-face	19	28	Clinicians and psychologists	Correct medication intakes	28
Pradeep et al., 2014 India [52]	Cluster RCT	24	260	122	138	NR	100.00	MDD + PD, social phobia or GAD	Women ≥ 18 years MDD (DSM-IV-TR)	Education + support	Face-to-face	2	4	Health workers	Duration of compliance (days)	28
Richards et al., 2016 UK [53]	Cluster RCT	52	581	276	305	NR	71.94	Depressive episode	≥ 18 years Depressive episode (ICD-10)	CCM	Face-to-face	6–12	≥ 3	Trained care managers, GPs and mental health worker	Self-report	16 52
Rickles et al., 2006, 2005 USA [54, 55]	RCT	24	63	31	32	37.6 (17.15)^a^	84.10	Depressive symptoms	≥ 18 years BDI-II ≥ 16 Willingness to take AD	Education + monitoring	Telephone	3	3	Trained pharmacist	Medication intakes	12 24
Salkovskis et al., 2006 UK [56]	RCT	26	77	39	38	40.5 (NR)	81.82	Depressive disorder	17–70 year AD prescription	Self-help programme	Telephone	NR	6.5	GPs	Length of time medication	26
Simon et al., 2011 USA [58]	RCT	24	197	104	93	45.5 (NR)	72.12	Depressive disorder	≥ 18 years New AD No AD ≥ 270 days before Online messaging	Support	Telephone	4	4	GPs, psychiatrist and nurse	Using antidepressant for over 90 days	24
Simon et al., 2006 USA [57]	RCT	24	207	103	104	43.0 (21.21)^a^	65.00	MDD or PDD	≥ 18 years MDD or PDD New AD prescription	Support	Telephone	3	3	Nurses	Automated data on refill	12
Smit et al., 2005 Netherlands [60]	RCT	52	267	112	72	42.8 (19.39)^a^	63.20	MDD	18–70 years MDD (DSM-IV)	Education	Face-to-face + telephone	3	3	GPs	Correct medication intakes	12 24 36 52
				39						Education + psychiatric consultation		4	3	GPs and psychiatrist		
				44						Education + CBT		15	3	GPs and clinical psychologist		
Vannachavee, 2016 Thailand [61]	RCT	6	60	30	30	45.3 (22.70)^a^	84.00	MDD	≥ 18 years MDD (DSM-IV-TR) A new AD prescription Thai speaking	Educational, motivational and cognitive intervention	Face-to-face	4	1,5	Candidate master degree researcher and nurses	Self-Medication Intake Record Form	6
Vergouwen et al., 2009, 2005 Netherlands [62, 63]	Cluster RCT	26	211	101	110	43.0 (20.29)^a^	67.40	MDD	≥ 18 years MDD (DSM-IV)	Education + support + active participation in treatment process with discussion on AD	Mail + face-to-face	7 visits	6,5	GPs	Self-report + pill counts	10 26
Wiles et al., 2014, 2013 UK [65, 66]	RCT	52	469	234	235	49.6 (11.7)	72.30	MDD + PD, social phobia or GAD	18–75 years AD prescription Patients’ adherence to the prescribed AD BDI-II ≥ 14	CBT	Face-to-face	12–18	12	Trained CBT therapist	4-item MMAS (80%)	48
Wiles et al., 2008 UK [64]	RCT	16	25	14	11	45.3 (NR)	84		18–65 years Depressive disorder (ICD-10) AD ≥ 15 BDI-II Positive Morisky-Green-Levine test	CBT	Face-to-face	12–20	4	GPs, psychiatrist and psychologist	4-item MMAS (80%)	16
Yusuf et al., 2021 [68]	RCT	24	120	60	60	NR	81 (890.20)	MDD	≥ 18 years MDD (ICD-10) AD prescription	Education + support	Face-to-face + telephone	1 sessions + 6 telephone calls	6	Pharmacist	MMAS	24

^a^Own estimation, *AD* Antidepressant, *AG* Agoraphobia, *Base* Baseline, *CBT* Cognitive behavioural therapy, *CCM* Collaborative care model, *CG* Control group, *Cluster RCT* Cluster randomized controlled trials, *GAD* Generalized anxiety disorder, *GP* General practitioner, *IG* Intervention group, *m* months, *MDD* Major depressive disorder, *MMAS* Morisky Medication Adherence Scale, *N* total sample, *NA* Not applied, *NR* Not reported, *nRCT* non-randomized controlled, *PC* Panic disorder, *PDD* Persistent depressive disorder or Dysthymic Disorder, *Reminder APP* Medication reminder app, *SDM* Share decision making, *RCT* Randomized controlled trials, *w* weeks

Study size ranged from 19 to 12,919 participants, with a mean average of 526 per study. In the 46 studies, a total of 31,832 participants were recruited and 24,324 were finally assigned to intervention (RCT: 7,608; cluster-RCT: 3,470; nRCT: 13,147; cluster-nRCT: 99). The mean age of participants was 42.40 years (SD: 15.66) and 65.05% of them were female. Approximately 10% were lost in the follow-up, thus 2,404 patients completed the studies.

Most of the studies enrolled patients with depression at different levels of severity. However, five studies required a combination of major depressive disorder with panic disorder, social phobia or generalized anxiety disorder, or anxiety [34, 44, 45, 52, 65, 66].

All the studies assessed individual interventions and used usual care as comparator. In general, the number of sessions or contacts of the interventions ranged from 1 to 20. A total of 10 studies assessed the effects of the Collaborative Care Model (CCM) consisting of the following four elements of collaborative care: 1) a multi-professional approach to patient care; 2) a structured management plan, included either or both pharmacological and non-pharmacological interventions; 3) scheduled patient follow-ups to provide specific interventions, facilitate treatment adherence, or monitor symptoms or adverse effects; and 4) enhanced inter-professional communication. Five studies assessed the effects of interventions with only an educational focus while eight studies evaluated the effects of education and support, three of them used the RHYTHMS programme, a patient education programme which mails information directly to patients being treated with antidepressant medicines in a time-phased manner. Education was also added to Cognitive Behavioural Therapy (CBT), CBT and motivational interview, coaching, monitoring and psychiatric consultation. Psychotherapy was another type of included intervention; in particular, six studies used CBT, one study included short psychodynamic supportive psychotherapy and one study included interpersonal psychotherapy. Other types of interventions were shared decision-making, support, counselling, the use of medication reminder applications for mobile phones, Enhanced Care and Treatment Initiation and Participation, an intervention aimed at modifying factors such as psychological barriers, concerns about treatment, fear of antidepressants and misconceptions of depression treatment.

Intervention modalities included face-to-face meetings alone (18 studies) or in combination with telephone conversations (3 studies), leaflets (2 study), videotapes (2 studies), mails (1 study) or website. Eight studies used telephone-conversations and two studies used the same intervention in combination with mails and one study combined the same intervention with letters. Moreover, leaflets were used in three of the studies, while consultation of websites was included in two studies. Another intervention modality was the use of a smartphone (2 studies).

The intervention providers varied among studies: multidisciplinary teams (16 studies), primary care professionals -general practitioners, clinicians or internal medicine doctors- (8 studies), pharmacists (8 studies); psychiatrists, psychologists or therapists (5 studies), nurses (2 studies), research assistant (1 study), and health worker (1 study). In the remaining studies, the providers were required to deliver intervention (2 studies) or not reported (1 study).

All patients in the included studies were in the implementation phase of the adherence. Twenty-five studies provided short-term (ranged from 4 to 16 weeks), 22 studies provided mid-term (ranged from 20 to 36 weeks), and seven studies provided long-term (ranged from 48 to 76 weeks) outcomes. Both self-report and direct measures were used for assessing adherence. Approaches for subjectively assessed adherence included questionnaires, diaries and interviews, and approaches for objectively assessed adherence included electronic measures, pill count and plasma drug concentration.

### Risk of bias in the included studies

Out of the 41 RCTs identified, three were classified as having low risk of bias in all RoB 2.0 domains [34, 57, 70] (Table 2). In the remaining RCTs, the most common methodological concerns involved bias arising from the randomization generation and allocation concealment process (3 RCTs at high RoB) and bias in measurement of the outcome (6 at high RoB).

**Table 2:** Risk of bias of included RCTs

**Cluster-RCTs**							
**Study**	**Domains**						
	**Randomization process**		**Identification and recruitment of participants**	**Effect of assignment to intervention**	**Missing outcome data**	**Measurement of the outcome**	**Selection of the reported result**
Akerblad 2003 [26]	High		Low	Low	Low	Some concerns	Low
Chang 2014 [72]	Low		Low	Low	Low	Some concerns	Low
Keeley 2014 [38]	Low		Low	Low	Low	Some concerns	Some concerns
LeBlanc 2015 [41]	Unclear		Low	Some concerns	Low	Some concerns	Low
Pradeep 2014 [52]	Some concerns		Low	Low	Some concerns	Low	Low
Richards 2016 [53]	Low		Low	Low	Low	High	Low
Vergouwen 2009, 2005 [62, 63]	Low		Low	Low	Some concerns	Some concerns	Low
**Individually RCTs**							
**Study**	**Domains**						
	**Randomization process**			**Effect of assignment to intervention**	**Missing outcome data**	**Measurement of the outcome**	**Selection of the reported result**
Adler 2004 [25]	Low			Low	Low	High	Low
Aljumah & Hassali, 2015 [59]	Low			Some concerns	High	Low	Low
Al-Saffar 2008, 2005 [37, 48]	Low			Low	Some concerns	Some concerns	Low
Browne 2002 [70]	Low			Low	Low	Low	Low
Capoccia 2004 [71]	Some concerns			Low	Low	Some concerns	Low
De Jonghe 2001 [74]	Low			Some concerns	Low	Some concerns	Some concerns
Guo 2015 [67]	Some concerns			Low	Low	Some concerns	Some concerns
Hammonds 2015 [29]	Some concerns			Some concerns	Some concerns	Low	High
Interian 2013 [30]	Some concerns			Low	Low	Low	Low
John 2016 [31]	Low			Low	Some concerns	High	Some concerns
Katon 2002 [32]	Some concerns			Low	Some concerns	Some concerns	Some concerns
Katon 2001 [33]	Some concerns			Some concerns	Low	Some concerns	Low
Katon 1999 [34]	Low			Low	Low	Low	Low
Katon 1996 [35]	Some concerns			Some concerns	Low	Some concerns	Low
Katon 1995 [36]	Low			Low	Low	Some concerns	Low
Kutcher 2002 [40]	Low			Some concerns	High	Some concerns	Low
Perlis 2002 [75]	Some concerns			Low	Low	Some concerns	Low
Lin 2003 [42]	Some concerns			Low	Low	Low	Low
Lin 1999 [43]	Some concerns			Low	Some concerns	High	Low
Mantani 2017 [44]	Low			Low	Low	Some concerns	Low
Mundt 2001 [46]	Some concerns			Some concerns	Low	Some concerns	Low
Myers & Calvert, 1984 [49]	Some concerns			Low	Low	Some concerns	Low
Nwokeji 2012 [50]	High			Low	Low	Some concerns	Low
Perahia 2008 [51]	Some concerns			Low	Low	High	Low
Salkovskis 2006 [56]	Some concerns			Low	Some concerns	High	Some concerns
Rickles 2006, 2005 [54, 55]	Low			Low	High	Low	Low
Simon 2006 [57]	Low			Low	Low	Low	Low
Simon 2011 [58]	Low			Low	Low	Some concerns	Low
Smit 2005 [60]	High			Some concerns	Low	Low	Low
Vannachavee 2016 [61]	Some concerns			Low	Some concerns	Low	Low
Wiles 2014, 2013 [65, 66]	Low			Low	Some concerns	Some concerns	Low
Wiles 2008 [64]	Low			Low	Low	Some concerns	Low
Marasine, 2020 [69]	Low			Some concerns	Some concerns	Low	Low
Yusuf, 2021 [68]	Low			Some concerns	Some concerns	Low	Low

*High*, High risk of bias, *Low* Low risk of bias, *Unclear* Unclear risk of bias

*RCTs* Randomized controlled trials

For the five n-RTCs identified, risk of bias was generally low-to-moderate across all of them, all presenting risk of bias in at least three domains (Table 3).

**Table 3:** Risk of bias of included nRCTs

Study	Domains						
	**Bias due to confounding**	**Bias in selection of participants**	**Bias in classification of interventions**	**Bias due to deviations from intended interventions**	**Bias due to missing data**	**Bias in measurement of outcomes**	**Bias in selection of the reported result**
Desplenter et al., 2013 [27]	Moderate	Low	Low	Low	NI	Moderate	Moderate
Gervasoni et al., 2010 [28]	Serious	Low	Moderate	Low	NI	Low	Low
Myers and Calvert, 1976 [47]	NI	NI	Low	Low	Moderate	Moderate	Moderate
Klang et al., 2015 [39]	Moderate	NI	Low	Low	Moderate	Moderate	Moderate
Meglic et al., 2010 [45]	Moderate	Low	Moderate	Low	Moderate	Moderate	Moderate

*Serious* Serious risk of bias, *Moderate* Moderate risk of bias, *Low* Low risk of bias

*NI* No information, *nRCTs* non-randomized controlled trials

### Publication bias

**Fig. 2:**
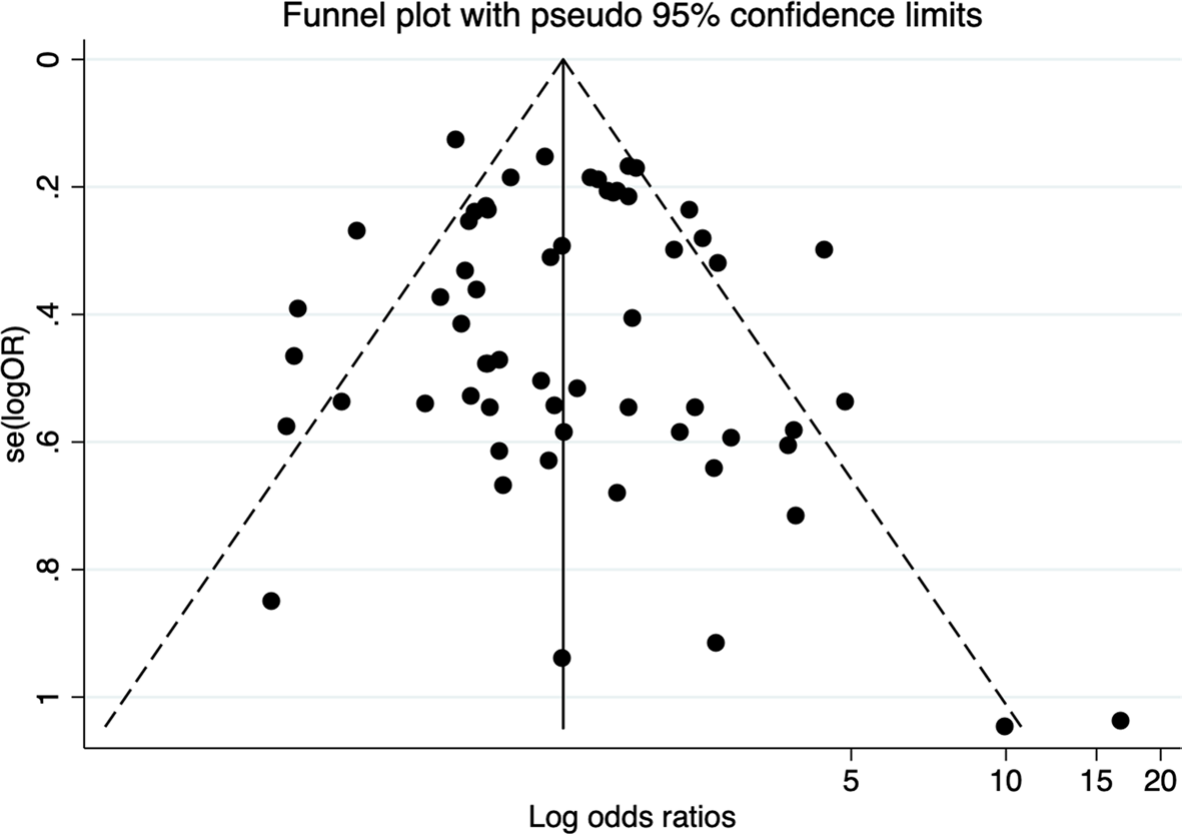
Funnel plot – Potential publication bias

No evidence of publication bias was found according to the funnel plot of the observed effect (Fig. 2) and the Egger’s regression test (*P* = 0.50).

### Synthesis of results

Results on adherence of the selected studies are available in the Supplementary Material (see Supplementary Table 2). Results of all meta-analyses and subgroup analysis are also available in the Supplementary Material (see Supplementary Tables 3 and 4).

**Fig. 3:**
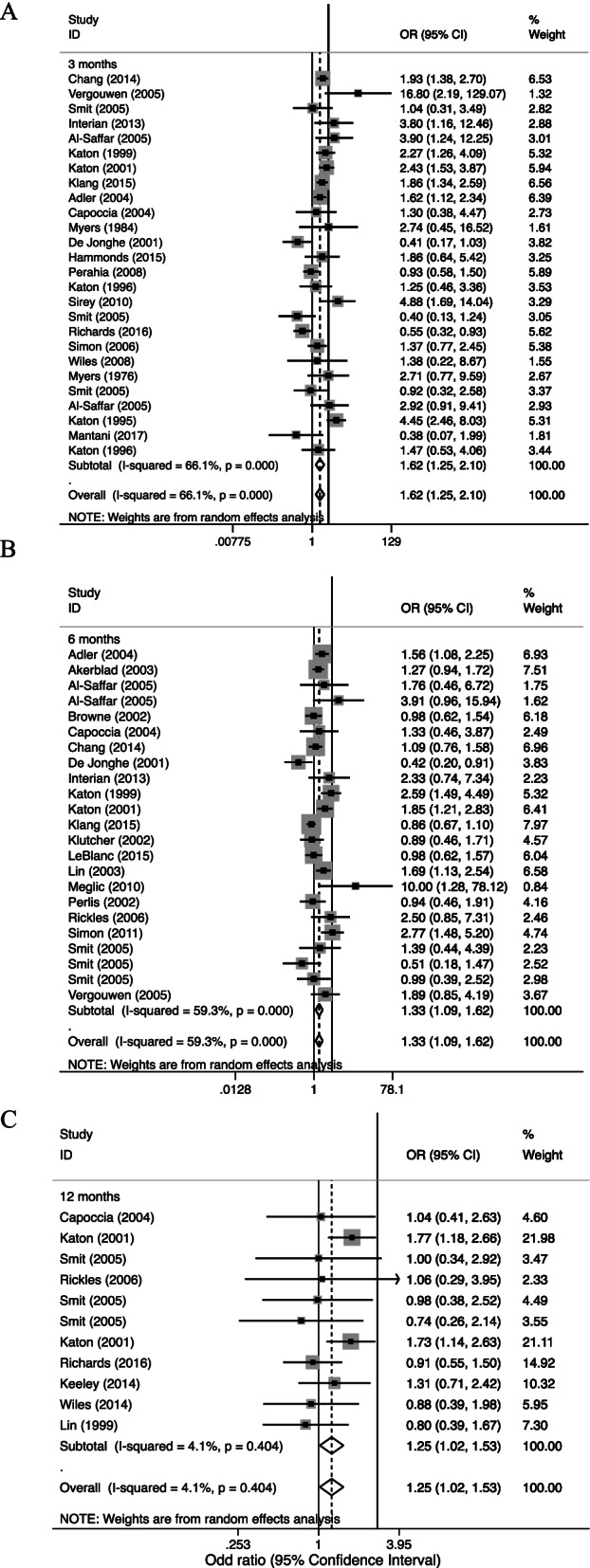
Forest plots for effect of intervention on adherence rate. Note: **A** at 6 months; **B** at 6 months; **C** at 12 months

Interventions aimed at improving the implementation phase of medication adherence in adults with depressive disorders had a positive effect on adherence outcome at 6 months after intervention compared with usual care (Odd ratio [OR] 1.33, 95% confidence interval [95% CI]: 1.09 to 1.62; *p* < 0.01) (Fig. 3). As anticipated, there was a moderate level of heterogeneity between studies (I^2^ = 59.30%).

In the patients of these studies, the overall trend for clinical improvement was observed to emerge at 3 months after intervention (OR 1.62, 95% CI: 1.25 to 2.10; *p* < 0.01) but the effect was attenuated at 12 months after intervention (OR 1.25, 95% CI: 1.02 to 1.53; I^2^ = 4.10%; *p* = 0.40) (Fig. 3). Substantial between-study heterogeneity was also found at 3 months (I^2^ = 66.10%).

### Causes of heterogeneity

Sufficient study-level data were available from 35 of the studies for the effect of the predictor variables to be entered into a subgroup or meta-regression analysis. Results of subgroup analysis and meta-regression are available in the Supplementary Material (see Supplementary Tables 3 and 4, respectively).

#### Diagnosis

Interventions aimed at improving adherence to medication when addressed to adults with depression at different levels of severity were associated with a significantly increased effect size (OR *Major depressive disorder or dysthymic disorder and anxiety studies* 2.77, 95% CI: 1.74 to 4.42; *p* < 0.01; OR *High risk for recurrent depression* 1.69, 95% CI: 1.13 to 2.54; *p* = 0.01; OR *Major depressive disorder or dysthymic disorder* 1.32, 95% CI: 1.08 to 1.61; *p* < 0.01; I^2^ = 35.80%). However, pooled effect sizes of studies on patients with depressive symptoms (OR, 2.50, 95% CI: 0.86 to 7.31; *p* = 0.29; I^2^ = NA%), depressive episode (OR, 0.88, 95% CI: 0.69 to 1.12; *p* = 0.29; I^2^ = 0%), and major depressive disorder with or without dysthymic disorder (OR, 0.68, 95% CI: 0.30 to 1.50; *p* = 0.29; I^2^ = 70.70%) were not statistically significant.

#### Type of intervention

In the case of CCM interventions, the pooled result showed a significant increase in adherence (OR 1.88, 95% CI: 1.40 to 2.54; *p* < 0.27; I^2^ = 23.00%) compared to the control group. However, statistically significant differences were not found for other specific forms of intervention (see Supplementary Table 3).

#### Providers of the intervention

A multi-professional approach to patient care involving at least one primary care provider and another health professional (e.g., nurse, psychologist, psychiatrist or pharmacist) was associated with an increased effect size (OR 1.73, 95% CI: 1.21 to 2.46; I^2^ = 53.70%). A non-multidisciplinary approach was not statistically significant (OR 1.15, 95% CI: 0.94 to 1.40; I^2^ = 42.90%).

#### Modality of intervention delivery

Effect sizes did not significantly differ by the modality of intervention delivery used (see Supplementary Table 3).

#### Other sources of heterogeneity

The number of intervention sessions was related to adherence (β, -0.08; 95% CI: -0.14 to -0.02). However, none of the other sources of heterogeneity investigated (age and gender of participants) had an effect.

### Cumulative meta-analysis of outcome at 6 months

**Fig. 4:**
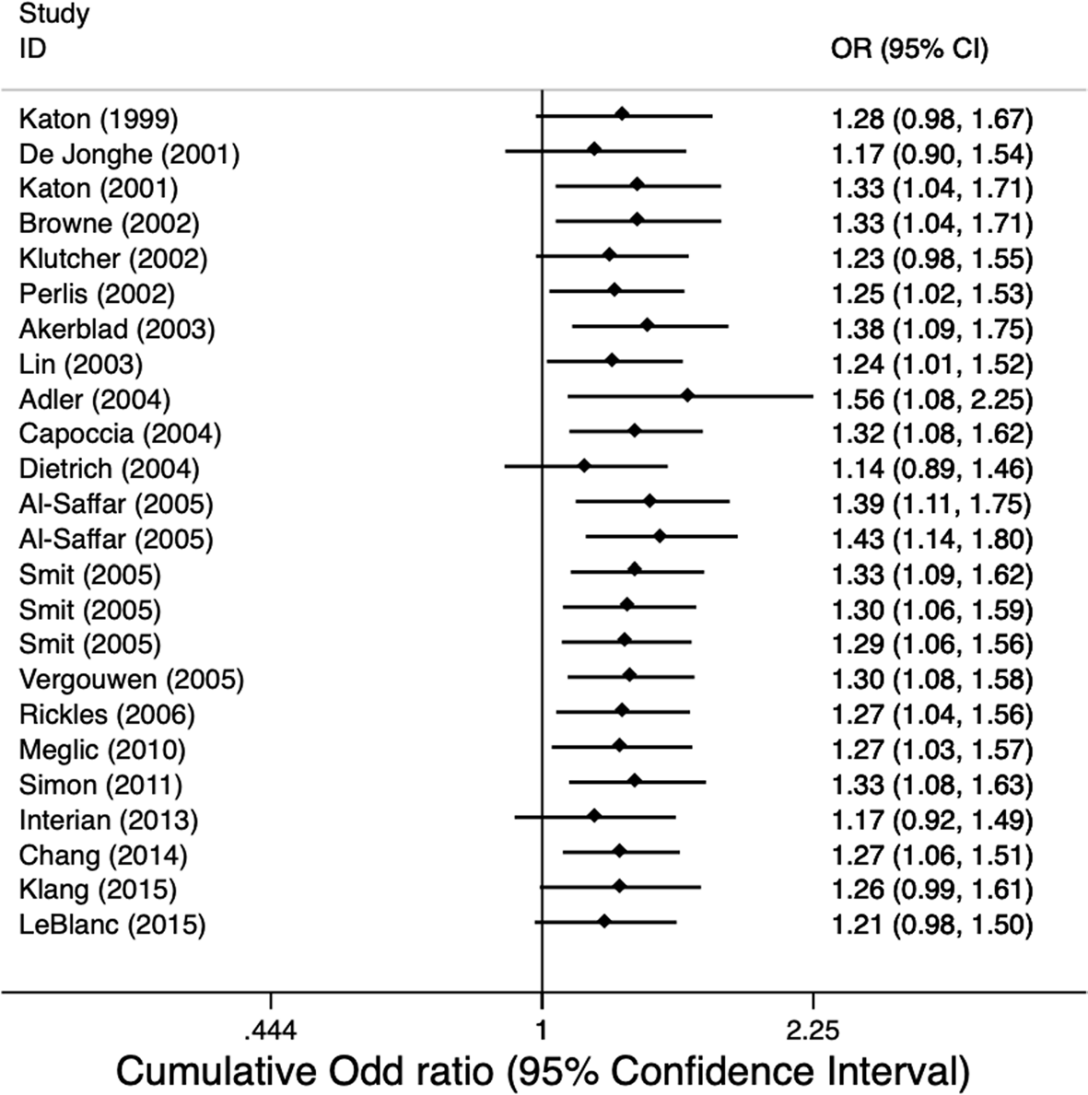
Cumulative meta-analysis of studies ordered by year of publication

When we assess interventions aimed at improving adherence to medication over time (Fig. 4), it is unclear whether earlier trials meeting the inclusion criteria demonstrated a high degree of heterogeneity or a high percentage of negative results. There is a sufficient body of evidence to demonstrate a reliable, consistent and statistically significant benefit of interventions aimed at improving adherence to medication over usual care. In general, the overall effect size has remained relatively stable within an effect size between OR 1.17 and 1.56.

## Discussion

Our findings support and confirm the notion that interventions aimed at improving adherence to medication among adults with depressive disorders are effective in improving outcomes in implementation phase of adherence in the studied patients, when these were analysed at 3 and 6 months after the intervention. The evidence, when given using cumulative meta-analysis, shows that further trials are unlikely to overturn this positive result. However, it is possible to appreciate a small decline in effect size over time.

The evidence shows that collaborative care is effective in improving adherence. In this respect, a multi-professional approach to patient care was more effective than primary or mental healthcare teams. This finding supports the idea that collaborative care might not only be clinically effective for symptom management in adults with depressive disorders [76, 77], but could also have a major effect on improving adherence to treatment [7]. This is in line with previous literature and suggests that multifaceted interventions targeting all dimensions that affect medication adherence problems, i.e., the patient, the healthcare provider and the health care delivery system, are more effective than single-component interventions to improve medication adherence [14, 15]. In fact, this positive effect of multicomponent interventions has also been observed in other psychiatric disorders [16, 17] and non-psychiatric pathologies [78]. Moreover, the number of intervention sessions was negatively related to adherence. A similar result has been observed in other studies of behaviour changes [79, 80]. Although the optimal number of intervention sessions is not clear, this a priori surprising result would support the usefulness of brief interventions or therapies to improve treatment adherence, however, it needs to be confirmed with more research.

Nevertheless, subgroup analyses indicate how other characteristics of the intervention may not help to enhance adherence. The modality of intervention and the provider profile were unrelated to effect size. Effect sizes also did not differ significantly by the modality of intervention delivery used (face-to-face vs. telephone, mails and/or website). Computer support systems, mobile technologies, web-based e-mail or telephone-based assistance can be used for improving adherence to medication [81, 82]. In this regard, these interventions may be available across different geographic areas and in different clinical settings [83].

Generally, it might be expected that patients with severe symptoms would have different treatment and support needs, and thus may profit from this type of interventions compared to patients with moderate or mild symptoms. However, the findings here do not show a clearly relationship between the severity of disease and adherence. Several interventions are effective in improving adherence outcomes among patients diagnosed with depression and anxiety at the same time. Although effectiveness is also demonstrated in the cases of patients at high risk of recurrent depression and in patients with major depressive disorder or dysthymic disorder, the results do not present such high values. Other patient characteristics such as age or gender were unconnected to adherence outcome.

The main limitation of the present review is the methodological differences between studies, mainly the diversity of both intervention procedures and severity and diagnosis of depressive disorder of participants, as well as the absence of an adequate psychopathological evaluation of the patients included in the studies. Interventions aimed at improving medication adherence among adults with emotional disorders have been designed with varying levels of intensity. Consequently, the review here found significant between-study heterogeneity. Subgroup and meta-regression analyses have been used to explore some of the issues related to the diversity of interventions (i.e.: type of intervention and providers) and patients’ characteristics (i.e.: severity of depression) that may influence the adherence result. Although, up to 770 determinants of adherence have been described in previous literature [84], only a few could be explored in this review. Although the prescribed antidepressant treatment has been shown to be a predictor of adherence [85, 86], most included studies did not report the specific antidepressant medicines that patients receive (Table 1). Moreover, there were studies that did not specify the patient´s phase of adherence, some of them because they were published before the publication of Vrijens et al. taxonomy [5]. However, after the evaluation based on the characteristics of the studies, we have determined that all patients in the included studies were in the implementation phase of the adherence. Finally, the exclusive reliance on English-language studies may not represent all the evidence. For this reason, we have also considered studies published in Spanish, however, limiting the systematic review to studies written in English and Spanish, which could introduce a language bias.

Despite all these limitations, our comprehensive systematic review provides an updated assessment of the effectiveness of different types of interventions aimed at improving medication adherence among adults with emotional disorders, supported by meta-analyses, using cumulative meta-analysis, assessing risk of bias of included studies, exploring important sources of heterogeneity and following rigorous and transparent methods compared to the previous systematic review [15].

The systematic review reported here shows that interventions aimed at improving short and medium-term adherence to medication among adults with depressive disorders are effective. Compared to short and medium-term adherence outcome, the available evidence on the effectiveness of long-term adherence is insufficient and supports the need for further research efforts.

## Supplementary Information

**Additional file 1: Supplementary Table 1.** Search strategy.

**Additional file 2: Supplementary Table 2.** Results on adherence in the included studies.

**Additional file 3: Supplementary Table 3.** Meta-Analyses of Adherence outcome and Subgroup Analyses.

**Additional file 4: Supplementary Table 4.** Meta-Regression Analyses (6 months).

## Data Availability

All data generated or analysed during this study are included in this published article and its supplementary information files.
