# Supplementary material for: Effectiveness of interventions to improve medication adherence in adults with depressive disorders: a meta-analysis
Source: BMC Psychiatry. 2022 Jul 20;22:487. doi: 10.1186/s12888-022-04120-w (PMC9301839; doi:10.1186/s12888-022-04120-w)
Supplement: Supplementary file 1 — Additional file 1: Supplementary Table 1. Search strategy. [file 12888_2022_4120_MOESM1_ESM.docx]

| **Supplementary Table 1.** Search strategy |
| --- |
| **Medline/PreMedline** |
| 1. Depression/ 2. Depressive Disorder/ 3. depressive disorder/ or depressive disorder, major/ 4. Mood Disorders/ 5. (depress* or mood? or affective disorder* or affective symptom* or depress* disorder* or dysphoria or endogenous depression or involutional depression or major depression or masked depression or melancholia or mood disorder or organic depression or recurrent brief depression or anaclitic depression or recurrent depression or treatment resistant depression or dysthymic disorder or reactive depression or agitated depression or atypical depression or sadness).ti,ab. 6. 1 or 2 or 3 or 4 or 5 7. Bipolar Disorder/ 8. Psychotic Disorders/ 9. (bipolar or bipolar disorder*).ti,ab. 10. (mania or manic*).ti,ab. 11. (Schizoaffective or schizo-affective).ti,ab. 12. 7 or 8 or 9 or 10 or 11 13. 6 not 12 14. Patient Compliance/ 15. Medication Adherence/ 16. Treatment Refusal/ 17. (adhere$ or non adherence or nonadherence or non-adherence or complian$ or non complian$ or non-complian$ or concordance or persistence or acceptance).mp. 18. management adherence.mp. 19. ((discontinu$ or abstention or abstain$ or stop$ or abandon$) adj4 therap$).tw. 20. ((discontinu$ or abstention or abstain$ or stop$ or abandon$) adj4 medic$).tw. 21. ((discontinu$ or abstention or abstain$ or stop$ or abandon$) adj4 treat$).tw. 22. Patient Dropouts/ 23. 14 or 15 or 16 or 17 or 18 or 19 or 20 or 21 or 22 24. Antidepressive Agents/ 25. Randomized Controlled Trials as Topic/ 26. randomized controlled trial/ 27. Random Allocation/ 28. Double Blind Method/ 29. Single Blind Method/ 30. clinical trial/ 31. clinical trial, phase i.pt. 32. clinical trial, phase ii.pt. 33. clinical trial, phase iii.pt. 34. clinical trial, phase iv.pt. 35. controlled clinical trial.pt. 36. randomized controlled trial.pt. 37. multicenter study.pt. 38. clinical trial.pt. 39. exp Clinical Trials as topic/ 40. or/25-39 41. (clinical adj trial$).tw. 42. ((singl$ or doubl$ or treb$ or tripl$) adj (blind$3 or mask$3)).tw. 43. PLACEBOS/ 44. placebo$.tw. 45. randomly allocated.tw. 46. (allocated adj2 random$).tw. 47. or/41-46 48. 40 or 47 49. case report.tw. 50. letter/ 51. historical article/ 52. or/49-51 53. 48 not 52 54. 13 and 23 and 24 and 53 55. limit 54 to humans 56. limit 55 to (english language or spanish) 57. remove duplicates from 56 |
| **EMBASE** |
| #1 'depression'/  #2 'major depression'/exp  #3 'mood disorder'/exp  #4 depress*:ti,ab OR mood?:ti,ab OR 'affective disorder*':ti,ab OR 'affective symptom*':ti,ab OR 'depress* disorder*':ti,ab OR dysphoria:ti,ab OR 'endogenous depression':ti,ab OR 'involutional depression':ti,ab OR 'major depression':ti,ab OR 'masked depression':ti,ab OR melancholia:ti,ab OR 'mood disorder':ti,ab OR 'organic depression':ti,ab OR 'recurrent brief depression':ti,ab OR 'anaclitic depression':ti,ab OR 'recurrent depression':ti,ab OR 'treatment resistant depression':ti,ab OR 'dysthymic disorder':ti,ab OR 'reactive depression':ti,ab OR 'agitated depression':ti,ab OR 'atypical depression':ti,ab OR sadness:ti,ab  #5 #1 OR #2 OR #3 OR #4  #6 'bipolar disorder'#  #7 'psychosis'  #8 bipolar:ti,ab OR 'bipolar disorder*':ti,ab  #9 mania:ti,ab OR manic*:ti,ab  #10 schizoaffective:ti,ab OR 'schizo-affective':ti,ab  #11 #6 OR #7 OR #8 OR #9 OR #10  #12 #5 NOT #11  #13 'patient compliance'  #14 'medication compliance'  #15 'treatment refusal'  #16 'patient dropout'  #17 adhere*:ti,ab,de OR 'non adherence':ti,ab,de OR nonadherence:ti,ab,de OR 'non-adherence':ti,ab,de OR complian*:ti,ab,de OR 'non complian*':ti,ab,de OR 'non-complian*':ti,ab,de OR concordance:ti,ab,de OR persistence:ti,ab,de OR acceptance:ti,ab,de  #18 'management adherence':ti,ab  #19 ((discontinu* OR abstention OR abstain* OR stop* OR abandon*) NEAR/4 therap*):ti,ab,de  #20 ((discontinu* OR abstention OR abstain* OR stop* OR abandon*) NEAR/4 medic*):ti,ab,de  #21 ((discontinu* OR abstention OR abstain* OR stop* OR abandon*) NEAR/4 treat*):ti,ab,de  #22 #13 OR #14 OR #15 OR #16 OR #17 OR #18 OR #19 OR #20 OR #21  #23 'antidepressant agent'  #24 'clinical trial'/de  #25 'randomized controlled trial'/de  #26 'controlled clinical trial'/de  #27 'multicenter study'/de  #28 'phase 3 clinical trial'/de  #29 'phase 4 clinical trial'/de  #30 'randomization'/exp  #31 'single blind procedure'/de  #32 'double blind procedure'/de  #33 'crossover procedure'/de  #34 'placebo'/de  #35 'randomi*ed controlled trial*':ti,ab  #36 rct:ti,ab  #37 (random* NEAR/2 allocat*):ti,ab  #38 'single blind*':ti,ab  #39 'double blind*':ti,ab  #40 ((treble OR triple) NEAR/1 blind*):ti,ab  #41 placebo*:ti,ab  #42 'prospective study'/de  #43 #24 OR #25 OR #26 OR #27 OR #28 OR #29 OR #30 OR #31 OR #32 OR #33 OR #34 OR #35 OR #36 OR #37 OR #38 OR #39 OR #40 OR #41 OR #42  #44 'case study'/de  #45 'case report':ti,ab  #46 'abstract report'/de OR 'letter'/de  #47 'conference paper'/it  #48 'conference abstract'/it  #49 'conference proceeding'/it  #50 'editorial'/it  #51 'note'/it  #52 #44 OR #45 OR #46 OR #47 OR #48 OR #49 OR #50 OR #51  #53 #43 NOT #52  #54 #12 AND #22 AND #23 AND #53  #55 #12 AND #22 AND #23 AND #53 AND [humans]/lim  #56 #12 AND #22 AND #23 AND #53 AND [humans]/lim AND ([english]/lim OR [spanish]/lim) |
| **PsycINFO** |
| S1 MA depression  S2 MA depressive disorder  S3 MA depressive disorder, major  S4 MA mood disorders  S5 TI ( (depress* or mood? or affective disorder* or affective symptom* or depress* disorder* or dysphoria or endogenous depression or involutional depression or major depression or masked depression or melancholia or mood disorder or organic depression or recurrent brief depression or anaclitic depression or recurrent depression or treatment resistant depression or dysthymic disorder or reactive depression or agitated depression or atypical depression or sadness) ) OR AB ( (depress* or mood? or affective disorder* or affective symptom* or depress* disorder* or dysphoria or endogenous depression or involutional depression or major depression or masked depression or melancholia or mood disorder or organic depression or recurrent brief depression or anaclitic depression or recurrent depression or treatment resistant depression or dysthymic disorder or reactive depression or agitated depression or atypical depression or sadness) )  S6 S1 OR S2 OR S3 OR S4 OR S5  S7 MA bipolar disorder  S8 MA psychotic disorders  S9 TI ( (bipolar or bipolar disorder*) ) OR AB ( (bipolar or bipolar disorder*) )  S10 TI ( (mania or manic*) ) OR AB ( (mania or manic*) )  S11 TI ( (Schizoaffective or schizo-affective) ) OR AB ( (Schizoaffective or schizo-affective) )  S12 S7 OR S8 OR S9 OR S10 OR S11  S13 S6 NOT S12  S14 MA patient compliance  S15 MA medication adherence  S16 MA treatment refusal  S17 DE (adhere* or "non adherence" or "nonadherence" or "non-adherence" or complian* or "non complian*" or "non-complian*" or concordance or persistence or acceptance)  S18 TI management adherence OR AB management adherence  S19 TX ((discontinu* or abstention or abstain* or stop* or abandon*) N4 therap*)  S20 TX ((discontinu* or abstention or abstain* or stop* or abandon*) N4 medic*)  S21 TX ((discontinu* or abstention or abstain* or stop* or abandon*) N4 treat*)  S22 TX patient dropouts  S23 S14 OR S15 OR S16 OR S17 OR S18 OR S19 OR S20 OR S21 OR S22  S24 MM "Antidepressant Drugs"  S25 S13 AND S23 AND S24  S26 MM "Clinical trials" OR MR ("Treatment Effectiveness Evaluation") OR TI ((randomi?ed n7 trial*) or ((single or doubl* or tripl* or treb*) and (blind* or mask*)) or (controlled n3 trial*) or (clinical n2 trial*)) OR AB ((randomi?ed n7 trial*) or ((single or doubl* or tripl* or treb*) and (blind* or mask*)) or (controlled n3 trial*) or (clinical n2 trial*)) OR KW ((randomi?ed n7 trial*) or ((single or doubl* or tripl* or treb*) and (blind* or mask*)) or (controlled n3 trial*) or (clinical n2 trial*))  S27 S25 AND S26 |
| **Social Science Citation Index and Science Citation Index** |
| # 1 774.313 TS= (depression or "depressive disorder" or "mood disorders")  # 2 TS= (depress* or mood? or "affective disorder*" or "affective symptom*" or "depress* disorder*" or dysphoria or "endogenous depression" or "involutional depression" or "major depression" or "masked depression" or "melancholia" or "mood disorder" or "organic depression" or "recurrent brief depression" or "anaclitic depression" or "recurrent depression" or "treatment resistant depression" or "dysthymic disorder" or "reactive depression" or "agitated depression" or "atypical depression" or sadness)  # 3 #2 OR #1  # 4 TS= (bipolar or "bipolar disorder*" or "psychotic disorder*" or mania or manic or "Schizoaffective" or "schizo-affective")  # 5 #3 not #4  # 6 TS= ("patient compliance" or "medication adherence" or "treatment refusal" or adhere* or "non adherence" or nonadherence or "non-adherence" or "complian*" or "non complian*" or "non-complian*" or concordance or persistence or acceptance or "management adherence")  # 7 TS= ((discontinu* or abstention or abstain* or stop* or abandon*) NEAR/4 therap*)  # 8 TS= ((discontinu* or abstention or abstain* or stop* or abandon*) NEAR/4 medic*)  # 9 TS= ((discontinu* or abstention or abstain* or stop* or abandon*) NEAR/4 treat*)  # 10 TS= "patient dropout*"  # 11 #10 OR #9 OR #8 OR #7 OR #6  # 12 TS= ("antidepress* agents" or "antidepress* medication" or "antidepress* drugs" or antidepress*)  # 13 #12 AND #11 AND #5  # 14 ((TS= clinical trial* OR TS=research design OR TS=comparative stud* OR TS=evaluation stud* OR TS=controlled trial* OR TS=follow-up stud* OR TS=prospective stud* OR TS=random* OR TS=placebo* OR TS=(single blind*) OR TS=(double blind*)))  # 15 #14 AND #13 |
| **CENTRAL** |
| #1 MeSH descriptor: [Depression] explode all trees  #2 MeSH descriptor: [Depressive Disorder] explode all trees  #3 MeSH descriptor: [Depressive Disorder, Major] explode all trees  #4 (depress* or mood? or affective disorder* or affective symptom* or depress* disorder* or dysphoria or endogenous depression or involutional depression or major depression or masked depression or melancholia or mood disorder or organic depression or recurrent brief depression or anaclitic depression or recurrent depression or treatment resistant depression or dysthymic disorder or reactive depression or agitated depression or atypical depression or sadness)  #5 #1 or #2 or #3 or #4  #6 MeSH descriptor: [Bipolar Disorder] explode all trees  #7 MeSH descriptor: [Psychotic Disorders] explode all trees  #8 (bipolar or bipolar disorder*)  #9 (mania or manic*)  #10 (Schizoaffective or schizo-affective)  #11 #6 or #7 or #8 or #9 or #10  #12 #5 not #11  #13 MeSH descriptor: [Patient Compliance] explode all trees  #14 MeSH descriptor: [Medication Adherence] explode all trees  #15 MeSH descriptor: [Treatment Refusal] explode all trees  #16 (adhere$ or non adherence or nonadherence or non-adherence or complian$ or non complian$ or non-complian$ or concordance or persistence or acceptance)  #17 management adherence  #18 ((discontinu$ or abstention or abstain$ or stop$ or abandon$) adj4 therap$)  #19 ((discontinu$ or abstention or abstain$ or stop$ or abandon$) adj4 medic$)  #20 ((discontinu$ or abstention or abstain$ or stop$ or abandon$) adj4 treat$)  #21 MeSH descriptor: [Patient Dropouts] explode all trees  #22 #13 or #14 or #15 or #16 or #17 #18 or #19 or #20 or #21  #23 MeSH descriptor: [Antidepressive Agents] explode all trees  #24 #12 and #22 and #23 |
