# Supplementary material for: Effectiveness of interventions to improve medication adherence in adults with depressive disorders: a meta-analysis
Source: BMC Psychiatry. 2022 Jul 20;22:487. doi: 10.1186/s12888-022-04120-w (PMC9301839; doi:10.1186/s12888-022-04120-w)
Supplement: Supplementary file 2 — Additional file 2: Supplementary Table 2. Results on adherence in the included studies. [file 12888_2022_4120_MOESM2_ESM.docx]

| **Supplementary Table 2.** Results on adherence in the included studies | | | | | | | | |
| --- | --- | --- | --- | --- | --- | --- | --- | --- |
| **Study** | **Measure** | **Period** | **Intervention** | | **Control** | | **OR (95%IC) / MD (95%IC) / P value** | **Intervention** |
|  |  |  | **Event / Mean (SD)** | **N** | **Event / Mean (SD)** | **N** |  |  |
| Adler et al., 2004 | Correct medication intakes | Baseline | 115 | 232 | 113 | 229 | 0.92 | CCM |
|  |  | 12 weeks | 141 | 232 | 112 | 229 | 0.024 |  |
|  |  | 24 weeks | 133 | 232 | 106 | 229 | 0.025 |  |
| Akerblad et al., 2003 | Self-report | 24 weeks | 236 | 366 | 200 | 339 | NR | Education and support |
|  | Serum levels | 24 weeks | 258 | 366 | 2019 | 339 | NR |  |
|  | Appointments kept | 24 weeks | 216 | 366 | 176 | 339 | NR |  |
|  | Composite index | 24 weeks | 155 | 366 | 124 | 339 | NR |  |
| Aljumah and Hassali, 2015 | MMAS | 12 weeks | 5.79 (1.89) | 110 | 5.04 (1.98) | 110 | 0.004 | Share decision making |
|  |  | 24 weeks | 5.99 (1.88) | 110 | 4.94 (1.94) | 110 | <0.0001 |  |
| Al-Saffar et al., 2008, 2005 | Self-report + Pill count | 8 weeks | 39 | 65 | 10 | 36 | NR | Education and support |
|  |  | 20 weeks | 43 | 54 | 10 | 21 | NR |  |
|  | Correct medication intakes | 6 weeks | 29 | 33 | 6 | 17 | NR |  |
|  | Self-report + Pill count | 8 weeks | 27 | 51 | 10 | 36 | NR | Education and counselling |
|  |  | 20 weeks | 25 | 42 | 10 | 21 | NR |  |
|  | Correct medication intakes | 6 weeks | 20 | 29 | 6 | 17 | NR |  |
| Browne et al., 2002 | Correct medication intakes | 24 weeks | 135 | 190 | 128 | 179 | NR | Interpersonal psychotherapy |
| Capoccia et al., 2004 | Self-report | 12 weeks | 35 | 41 | 27 | 33 | NR | CCM |
|  |  | 24 weeks | 32 | 41 | 24 | 33 | NR |  |
|  |  | 36 weeks | 20 | 41 | 22 | 33 | NR |  |
|  |  | 52 weeks | 24 | 41 | 19 | 33 | NR |  |
| Chang et al., 2014 | Currently taking | 12 weeks | 300 | 362 | 197 | 254 | 0.01 | Monitoring and feedback to physicians |
|  |  | 24 weeks | 299 | 380 | 189 | 284 | 0.01 |  |
|  | Never forgot to take in past 4 weeks | 12 weeks | 142 | 300 | 91 | 196 | 0.66 |  |
|  |  | 24 weeks | 153 | 299 | 92 | 188 | 0.42 |  |
|  | Never missed medication in last 4 weeks in response to feeling better | 12 weeks | 258 | 300 | 170 | 197 | 0.51 |  |
|  |  | 24 weeks | 250 | 299 | 164 | 189 | 0.85 |  |
| De Jonghe et al., 2001 | Pharmacotherapy dropout rate | 8 weeks | 4 | 72 | 3 | 57 | 0.942 | Short Psychodynamic Supportive Psychotherapy |
|  |  | 16 weeks | 9 | 72 | 15 | 57 | 0.045 |  |
|  |  | 24 weeks | 16 | 72 | 23 | 57 | 0.026 |  |
| Desplenter et al., 2013 | MMAS** | 4 weeks | 3.56 (0.58) | 27 | 3.21 (1.06) | 24 | NR | Counselling |
|  |  | 12 weeks | 3.41 (0.80) | 22 | 3.52 (0.9) | 23 | NR |  |
|  |  | 4 weeks | 3.43 (.084) | 37 | 3.21 (1.06) | 24 | NR | Tailoring counselling |
|  |  | 12 weeks | 3.29 (0.84) | 34 | 3.52 (0.9) | 23 | NR |  |
| Gervasoni et al., 2010 | AD plasma level | 2 weeks | 24  (1-87) | 81 | 24 (7-126) | 50 | 0.88 | Monitoring and motivational support |
| Hammonds et al., 2015 | Correct medication intakes | 4 weeks | 20 | 30 | 14 | 27 | 0.95 to 12.97 | Medication reminder app |
| Interian et al., 2013 | Pill Count | 5 weeks | 19 | 26 | 10 | 24 | NR | Motivational Enhancement Therapy |
|  |  | 20 weeks | 14 | 26 | 8 | 24 | NR |  |
| John et al., 2016 | Correct medication intakes | 6 weeks | 5 | 17 | 8 | 22 | NR | Educational module |
| Katon et al., 2001 | Automated data on refill | 12 weeks | 157 | 194 | 122 | 192 | NR | CCM |
|  |  | 24 weeks | 140 | 194 | 112 | 192 | NR |  |
|  |  | 36 weeks | 133 | 194 | 107 | 192 | NR |  |
|  |  | 52 weeks | 123 | 194 | 95 | 192 | NR |  |
| Katon et al., 1999 | Automated data on refill | 4 weeks | 88 | 114 | 79 | 114 | 0.24 | CCM |
|  |  | 12 weeks | 90 | 114 | 71 | 114 | 0.02 |  |
|  |  | 24 weeks | 83 | 114 | 58 | 114 | 0.002 |  |
| Katon et al., 1996 | Automated data on refill | 4 weeks | 21 | 31 | 20 | 34 | 0.46 | CCM |
|  |  | 12 weeks | 19 | 31 | 19 | 34 | 0.55 |  |
| Katon et al., 1995 | Automated data on refill** | 4 weeks | 95 | 108 | 56 | 109 | NR | CCM |
|  |  | 12 weeks | 84 | 108 | 48 | 109 | NR |  |
| Keeley et al., 2014 | Filled prescription | 52 weeks | 53 | 85 | 48 | 86 | 0.63 | Motivational Interviewing |
| Klang et al., 2015 | Correct medication intakes | 4 weeks | 123 | 173 | 7256 | 12746 | <0.0001 | Pharmacist adherence support |
|  |  | 24 weeks | 95 | 173 | 294 | 1934 | <0.0001 |  |
| Klutcher et al., 2002 | Pill count | NR | 109 | 131 | 117 | 138 | 0.7 | Education and support (programme RHYTHMS) |
| LeBlanc et al., 2015 | Automated data on refill | 24 weeks | 96 | 158 | 85 | 139 | 0.25 | SDM |
| Lin et al., 2003 | Self-report (80%) | 36 weeks | 122 | 194 | 96 | 192 | 1.91 (1.37 to 2.65) | CBT, motivational interviewing and education |
| Lin et al., 1999 | Self-reported (80%) | 76 weeks | 31 | 63 | 29 | 53 | 0.68 | CCM |
|  | Adequate pharmacotherapy | 76 weeks | 47 | 63 | 33 | 53 | 0.22 |  |
| Mantani et al., 2017 | Discontinuation of protocol antidepressant treatment | 9 weeks | 16 | 81 | 14 | 83 | NR | Smartphone CBT |
|  | Discontinuation of any antidepressant therapy | 9 weeks | 5 | 81 | 2 | 83 | NR |  |
|  | Escitalopram dosage | 9 weeks | 48 | 81 | 49 | 83 | NR |  |
|  | Sertraline dosage | 9 weeks | 25 | 81 | 29 | 83 | NR |  |
| Marasine et al., 2020 | MMAS | 8 weeks | 1 (2) | 98 | 2 (2) | 98 | <0.01 | Education and counselling |
|  |  | 16 weeks | 1 (2) | 98 | 2 (2) | 98 | <0.01 |  |
| Meglic et al., 2010 | Medication adherence | 24 weeks | 10 | 12 | 3 | 9 | 0.03 | CCM |
| Mundt et al., 2001 | Medication days | 30 weeks | 100 (66) | 89 | 102 (68) | 82 | NR | Education and support (programme RHYTHMS) |
| Myers and Calvert, 1976 | Correct medication intakes | NR | 41 | 46 | 34 | 43 | NR | Education |
| Myers and Calvert, 1984 | Correct medication intakes | 3 weeks | 28 | 32 | 24 | 32 | NR | Side effects information |
|  |  |  | 30 | 33 | 24 | 32 | NR | Beneficial effects information |
|  |  | 6 weeks | 26 | 28 | 19 | 23 | NR | Side effects information |
|  |  |  | 26 | 27 | 2719 | 23 | NR | Beneficial effects information |
| Nwokeji et al., 2012 | % of days covered | 52 weeks | 67.2 (23.8) | 101 | 63.6 (27.0) | 65 | 0.36 | Enhanced care |
| Perahia et al., 2008 | Pill count | 2 weeks | 467 | 477 | 463 | 481 | NR | Education |
|  |  | 6 weeks | 456 | 477 | 458 | 481 | NR |  |
|  |  | 12 weeks | 439 | 477 | 445 | 481 | NR |  |
| Perlis et al., 2002 | Correct medication intakes | 28 weeks | 42 | 66 | 43 | 66 | NR | CBT |
| Pradeep et al., 2014 | Duration of compliance (days) | 28 weeks | 77.7 (72.8) | NR | 23.31 (26.59) | NR | < 0.01 | Education and support |
| Richards et al., 2016 | Self-report** | 16 weeks | 192 | 230 | 248 | 275 | NR | CCM |
|  |  | 52 weeks | 200 | 235 | 227 | 263 | NR |  |
| Rickles et al., 2006, 2005 | Medication intakes** | 12 weeks | 23 | 28 | 26 | 32 | > 0.05 | Education and monitoring |
|  |  | 24 weeks | 20 | 28 | 16 | 32 | ≤ 0.05 |  |
| Salkovskis et al., 2006 | Length of time medication | 26 weeks | 32.3 (NR) | 39 | 28.8 (NR) | 38 | >0.02 | Self-help programme |
| Simon et al., 2011 | Using antidepressant | 24 weeks | 86 | 106 | 62 | 102 | 0.001 | Support |
| Smit et al., 2005 | Correct medication intakes | 12 weeks | 71 | 102 | 46 | 64 | NR | Education |
|  |  |  | 25 | 34 | 46 | 64 | NR | Education + psychiatric consultation |
|  |  |  | 20 | 40 | 46 | 64 | NR | Education + CBT |
|  |  | 24 weeks | 60 | 102 | 38 | 64 | NR | Education |
|  |  |  | 23 | 34 | 38 | 64 | NR | Education + psychiatric consultation |
|  |  |  | 17 | 40 | 38 | 64 | NR | Education +CBT |
|  |  | 36 weeks | 48 | 102 | 36 | 64 | NR | Education |
|  |  |  | 19 | 34 | 36 | 64 | NR | Education + psychiatric consultation |
|  |  |  | 18 | 40 | 36 | 64 | NR | Education + CBT |
|  |  | 52 weeks | 53 | 102 | 33 | 64 | NR | Education |
|  |  |  | 17 | 34 | 33 | 64 | NR | Education + psychiatric consultation |
|  |  |  | 18 | 40 | 33 | 64 | NR | Education + CBT |
| Simon et al., 2006 | Automated data on refill | 12 weeks | 63 | 98 | 55 | 97 | 0.17 | Support |
| Vannachavee et al., 2016 | Self-Medication Intake Record Form | 6 weeks | 41.17 (2.87) | 30 | 22.58 (17.07) | 26 | < 0.05 | Educational, motivational and cognitive intervention |
| Vergouwen et al., 2009, 2005 | Self-report + pill counts | 10 weeks | 86 | 87 | 87 | 104 | 0.4 to 2.2 | Education, support, and active participation in treatment |
|  |  | 26 weeks | 68 | 79 | 72 | 94 | 0.4 to 1.6 |  |
| Wiles et al., 2014, 2013, 2008 | MMAS (80%) | 48 weeks | 160 | 173 | 168 | 180 | −6.2 to 4.5 | CBT |
| Wiles et al., 2008 | MMAS (80%) | 16 weeks | 11 | 14 | 8 | 11 | NR | CBT |
| Yusuf et al., 2021 | MMAS | 12 weeks | 7.46 (1.34) | 52 | 5.53 (2.03) | 49 | <0.01 | Education and counselling |
|  |  | 24 weeks | 9.15 (1.62) | 52 | 5.22 (1.90) | 49 | <0.01 |  |

**: data sent by email by authors; CBT: cognitive behavioural therapy; CCM: collaborative care model; MD: mean difference; MMAS: Morisky Medication Adherence Scale; NR: not reported; OR: Odds ratio; SD: standard deviation
