# Supplementary material for: Effectiveness of interventions to improve medication adherence in adults with depressive disorders: a meta-analysis
Source: BMC Psychiatry. 2022 Jul 20;22:487. doi: 10.1186/s12888-022-04120-w (PMC9301839; doi:10.1186/s12888-022-04120-w)
Supplement: Supplementary file 3 — Additional file 3: Supplementary Table 3. Meta-Analyses of Adherence outcome and Subgroup Analyses. [file 12888_2022_4120_MOESM3_ESM.docx]

| **Supplementary Table 3.** Meta-Analyses of Adherence outcome and Subgroup Analyses | | | | |
| --- | --- | --- | --- | --- |
| **META-ANALYSES AND SUBGROUP ANALYSIS** | | | | |
|  | **N** | **OR** | **95% CI** | **I^2^ (%)** |
| **Follow up** | | | | |
| Total | 34 | 1.40 | 1.22 to 1.61 | 60.50 |
| Sensitivity analysis* | 33 | 1.38 | 1.20 to 1.58 | 60.60 |
| 3 months | 23 | 1.62 | 1.25 to 2.10 | 66.10 |
| Sensitivity analysis* | 22 | 1.60 | 1.23 to 2.01 | 67.20 |
| 6 months | 21 | 1.33 | 1.09 to 1.62 | 59.30 |
| Sensitivity analysis* | 20 | 1.33 | 1.10 to 1.60 | 58.00 |
| 12 months | 7 | 1.25 | 1.02 to 1.53 | 4.10 |
| **Diagnoses** (6 months) | | | | |
| MDD or PDD and anxiety | 3 | 2.77 | 1.74 to 4.42 | 0 |
| Sensitivity analysis* | 2 | 2.58 | 1.60 to 4.17 | 0 |
| MDD or PDD | 12 | 1.32 | 1.08 to 1.61 | 35.80 |
| MDD with or without PDD | 2 | 0.68 | 0.30 to 1.50 | 70.70 |
| High risk for recurrent depression | 1 | 1.69 | 1.13 to 2.54 | NA |
| Depressive episode | 2 | 0.88 | 0.69 to 1.12 | 0 |
| Depression symptoms | 1 | 2.50 | 0.86 to 7.31 | NA |
| **Type of intervention** (6 months) | | | | |
| CBT | 3 | 1.06 | 0.55 to 2.05 | 63.20 |
| CCM | 5 | 1.88 | 1.40 to 2.54 | 23.00 |
| Sensitivity analysis* | 4 | 1.80 | 1.41 to 2.30 | 0 |
| Counselling | 1 | 3.90 | 0.96 to 15.94 | NA |
| Education | 1 | 0.98 | 0.38 to 2.53 | NA |
| Education and monitoring | 1 | 2.50 | 0.86 to 7.31 | NA |
| Education and support | 5 | 1.28 | 0.99 to 1.64 | 0 |
| Enhance care | 1 | 2.33 | 0.74 to 7.34 | NA |
| Interpersonal psychotherapy | 1 | 0.98 | 0.62 to 1.53 | NA |
| Share Decision Making | 1 | 0.98 | 0.62 to 1.57 | NA |
| Short Psychodynamic Supportive Psychotherapy | 1 | 0.42 | 0.20 to 9.10 | NA |
| Support | 2 | 1.49 | 0.47 to 4.69 | 91.40 |
| **Modality of intervention** (6 months) | | | | |
| Mail and website | 2 | 3.15 | 0.68 to 14.63 | 59.70% |
| Face-to-face | 12 | 1.23 | 0.94 to 1.59 | 61.40 |
| Telephone | 6 | 1.38 | 1.01 to 1.88 | 45.70 |
| **Providers of the intervention** (6 months) | | | | |
| Multidisciplinary team | 8 | 1.73 | 1.21 to 2.46 | 53.70 |
| Sensitivity analysis* | 7 | 1.66 | 1.18 to 2.33 | 51.60 |
| Non-multidisciplinary team | 14 | 1.15 | 0.94 to 1.40 | 42.90 |

*: excluding non-randomized controlled trials

CBT: cognitive behavioural therapy sessions; CI: confidence interval; CCM: collaborative care model; MDD: [Major depressive disorder](https://en.wikipedia.org/wiki/Major_depressive_disorder); PDD: Persistent Depressive Disorder or Dysthymic Disorder; N: number of studies included in meta-analysis; OR: Odds ratio.
