# Supplementary material for: Effectiveness of interventions to improve medication adherence in adults with depressive disorders: a meta-analysis
Source: BMC Psychiatry. 2022 Jul 20;22:487. doi: 10.1186/s12888-022-04120-w (PMC9301839; doi:10.1186/s12888-022-04120-w)
Supplement: Supplementary file 4 — Additional file 4: Supplementary Table 4. Meta-Regression Analyses (6 months). [file 12888_2022_4120_MOESM4_ESM.docx]

| **Supplementary Table 4.** Meta-Regression Analyses (6 months) | | | | | |
| --- | --- | --- | --- | --- | --- |
|  | **Coefficient** | **SE** | **t** | **P-value** | **95% CI** |
| **Age** | -0.11 | 0.07 | -1.55 | 0.14 | -0.27 to 0.04 |
| **Gender** | 0.02 | 0.02 | 1.06 | 0.30 | -0.02 to 0.07 |
| **Number of sessions** | -0.08 | 0.03 | -2.64 | 0.02 | -0.14 to -0.02 |

CI: confidence interval; SE: standard error
